# Correlates of Morbidity and Mortality in Severe Necrotizing Pancreatitis

**DOI:** 10.5402/2012/215193

**Published:** 2012-07-09

**Authors:** K. M. Pal, Pashtoon Murtaza Kasi, Mohammad Tayyeb, S. M. Faisal Mosharraf, Zafar Fatmi

**Affiliations:** ^1^Department of Surgery, Aga Khan University, Karachi 74800, Pakistan; ^2^Department of Radiology, Aga Khan University, Pakistan; ^3^Department of Community Health Sciences, Aga Khan University, Pakistan

## Abstract

Acute severe pancreatitis is associated with a high morbidity and mortality and frequently is accompanied by underlying pancreatic parenchymal necrosis. Patients with pancreatic necrosis must be identified, because the morbidity and mortality rate in this subgroup is much higher. Our objective was to compare the clinical outcomes of these patients based on the degree of pancreatic necrosis. A total of 35 patients were noted to have pancreatic necrosis. These were divided into 2 groups based on extent of necrosis: group A had less than 50% necrosis and group B had more than 50% necrosis. The rate of mortality (5% versus 40%) was significantly higher in group B. The rate of organ dysfunction also rose along with the rates of other morbidities and variables that were related to a patient's hospital stay. Only APACHE II significantly correlated with the degree of necrosis, wherein the chances of substantial necrosis rose by 20% with each unit increase of APACHE II score. APACHE II Score could be employed and studied further prospectively to help identify patients with pancreatic necrosis.

## 1. Introduction

Acute pancreatitis is an inflammatory process that develops from damage to pancreatic acinar cells, which is caused by inappropriate activation of digestive enzymes within the cells. The mechanisms by which diverse etiological factors initiate an attack are unclear.

The wide range of clinical presentations is based on the extent and severity of the inflammatory response. From a mild event that is confined to the gland to necrosis of the pancreas with attendant multiorgan dysfunction, increasing severity is associated with increased morbidity and mortality [1, 2]. Numerous approaches to estimate the severity of an episode have been used, from clinical estimation and biochemical markers to multivariable scoring systems. A shortcoming of these methods is their inability to assess the extent of injury to the pancreas and peripancreatic tissues.

It is imperative that we identify patients with pancreatic necrosis, because morbidity and mortality rates in this subgroup are much higher [3, 4].

For the diagnosis of pancreatic parenchymal necrosis, intravenous contrast-enhanced CT scan is the ideal imaging method [5, 6]. The accepted criteria for the diagnosis of pancreatic necrosis on CT are focal or diffuse zones of nonenhanced pancreatic parenchyma, visualized during an examination with intravenous administration of contrast material.

In 1985, Balthazar et al. were the first to grade severity of pancreatitis based on CT findings [7]. Pancreatic tissue that has undergone necrosis typically encompasses the body or tail and shows decreased or no enhancement on CT and is surrounded by normally enhancing pancreatic tissue [8].

The focus of our study was to compare the predictive value of the Acute Physiological Assessment and Chronic Health Evaluation (APACHE II) system with CT-visualized extent of pancreatic injury in severe necrotizing pancreatitis.

## 2. Methods 

### 2.1. Data Collection

This report is a retrospective, descriptive case series. Patients who were admitted to the hospital between January 1999 and June 2006 with a diagnosis of acute pancreatitis were identified through the medical records system, using the ICD 9 coding. The medical records of all patients with documented pancreatic necrosis were then reviewed. Data was collected using a standardized questionnaire.

The percentage of pancreatic parenchymal necrosis was calculated by an independent review of the CT scans by a single radiologist (FM). Based on the extent of pancreatic necrosis on the CT scan, the patients were divided into 2 groups (group A patients, having less than 50% necrosis, and group B patients, having more than 50% necrosis). The APACHE II score was calculated from the medical records. Patients with incomplete records or missing CT scans were excluded from the study.

Cardiovascular dysfunction was defined as hypotension that required vasoactive medication; renal dysfunction as serum creatinine levels greater than 2 mg/dL; and respiratory dysfunction as the need for mechanical ventilation or PaO_2_ levels of less than 60 mmHg.

Data from reports of any cultures from surgery or fine needle aspirates (FNAs) were also collected. Infected pancreatic necrosis was defined as the presence of microorganisms in either culture. Other infections were not included in the current study.

Patients who died during the hospital stay were included in the mortality statistics.

### 2.2. Study Design

Clinical outcomes were compared between groups A (minimal necrosis, i.e., <50%) and B (substantial necrosis >50%). Also, factors were compared between survivors and nonsurvivors using univariate and multivariate analysis.

### 2.3. Data Collection and Statistical Analysis

A database was developed using Microsoft Access 2000, and the results were imported into SPSS, version 13.0. Frequencies, percentages, means, and standard deviations were computed when it was appropriate. The chi-square or Fisher's exact test was used to compare categorical variables while Student's *t*-test was used to compare continuous variables between the two groups. A double-sided *P *value of less than 0.05 was considered to be statistically significant. Parameters that differed in survivors and nonsurvivors by univariate analysis with a *P *value of 0.25 or less were entered into the logistic regression model, using mortality as the dependent variable, to identify factors that were independently related to mortality.

## 3. Results

A total of 1225 patients with a diagnosis of acute pancreatitis were admitted to the hospital during this period. 315 patients had an abdominal CT scan. Of them, 48 patients experienced pancreatic necrosis. Due to incomplete data or missing CT scans, 13 patients were excluded from further review.

### 3.1. Age and Sex Distribution

There were 19 (54.3%) males and 16 (45.7%) females. The mean age was 51.6 years, and the standard of deviation was 14.6 years (range 28–77 years).

### 3.2. Type of Admission

26 (74.2%) patients were admitted from the emergency room, 1 (2.9%) was an inpatient, and 7 (20%) were transferred from another hospital. Information was unavailable for 1 patient.

### 3.3. History and Cause of Pancreatitis

The cause was identified as biliary and alcohol-related pancreatitis in 19 (54.3%) and 4 (11.4%), respectively. The cause was unknown in the remaining 12 patients (34.3%). Four patients (11.4%) had a prior known history of pancreatitis.

### 3.4. Percentage Necrosis on CT Scan

Of the 35 patients, 20 (57.1%) had less than 50% necrosis and 15 (42.9%) had more than 50% pancreatic necrosis. The remaining analysis concerns these 2 groups (A and B, resp.).

### 3.5. Comparison of Characteristics between Groups A and B

There was no significant difference in sex (*P*  value—0.315) or age distribution in the groups (A—50.1 ± 15.0; and B—53.7 ± 14.0 years (*P*  value—0.480)). The type of admission (i.e., ER, inpatient, or from another hospital) also was similar between groups (*P*  value—0.467).

The prevalence of comorbidities (HTN, DM, IHD, COPD, and others) was comparable (*P*  value > 0.5). Timing of the CT scan also was similar, wherein the overall mean ± s.d. was 5.4 ± 4.7 days.

### 3.6. Rate of Mortality and Organ Dysfunction (Table 1)

**Figure 1:**
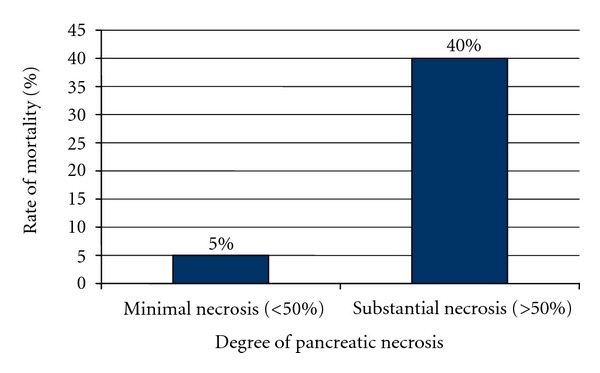
Rates of mortality in relation to the degree of pancreatic necrosis.

Seven patients died due to necrotizing pancreatitis. As shown in Table 1, 1 patient died in group A, and 6 patients (40%) died in group B. The rate of mortality was significantly higher in group B (*P*  value —0.027; Figure 1).

**Figure 2:**
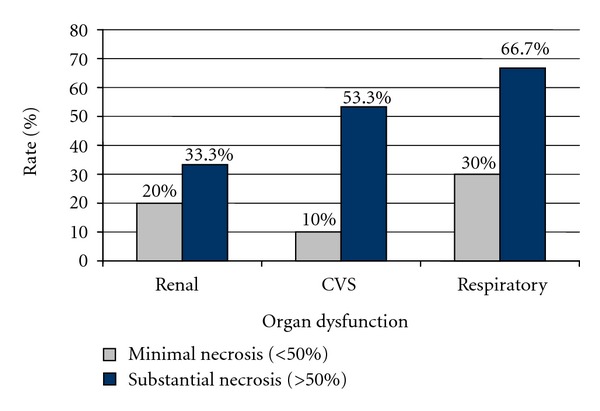
Rates of organ dysfunction in relation to the degree of pancreatic necrosis

The proportion of individuals who suffered from organ dysfunction also rose as the extent of necrosis increased (Table 1 and Figure 2).

### 3.7. Hospital Stay and Rate of Other Morbidities (Table 2)

Nearly half of the patients in group B required a stay in the ICU during their admission (7.86 ± 14.4 days). In contrast, only 2 patients from group A had to be transferred to the ICU (0.9 ± 3.39 days).

The hospital stay, the stay in the special care unit, and the duration that the patient had to be maintained NPO also were protracted as the extent of necrosis increased (Table 2).

Formation of pseudocysts was observed more frequently in group B. Surgery for pancreatic necrosis (necrosectomy) was performed in 5 patients, 2 of whom died (both of them had more than 50% pancreatic necrosis).

Infection in the pancreas (FNA or postoperative tissue) was present in 6 patients. *E. coli* grew in 4 of 6 cases, *Enterobacter* in 2 of 6 cases, *S*. *aureus* in 2 of 6 cases, *Enterococcus* in 1 of 6 cases, *Acinitobacter* in 1 of 6 cases, *Pseudomonas* in 1 of 6 cases, and *Citrobacter* Freundii in 1 of 6 cases. Of the 6 patients, 4 had less than 50% necrosis, and 2 had more than 50% necrosis. Both of the latter cases were offered surgery and died during admission.

Secondary diabetes mellitus developed in 6 patients, 4 of whom had more than 50% necrosis. Pancreatic fistula developed in 4 patients, 2 of whom were in group A and 2 in group B. Wound-related complications were noted in 3 patients, 2 of whom were in group B (1 in group A).

Readmission rates were higher in group B (60%) compared with group A (33.3%). These rates excluded persons who either died or were lost to follow-up.

### 3.8. Ranson, APACHE II, and Extent of Necrosis (Table 3)

There was no significant difference in the Ranson score between the 2 groups. APACHE II scores, however, were much higher for patients with greater necrosis (*P*  value 0.018).

### 3.9. Factors Predicting Outcome

In the univariate analysis, the statistically significant factors (*P*  value < 0.05) that correlated with mortality were the APACHE II score at admission, presence of CVS dysfunction, renal dysfunction, and substantial necrosis (>50%). Presence of infection, the need for surgery, Ranson score, and age had *P *values of less than 0.25. 

In the final model, the factors that had potential importance in predicting mortality were entered into the binary logistic regression analysis, using death as the outcome variable. The variables were age, APACHE II score at admission, the presence of substantial necrosis, presence of CVS dysfunction, and presence of renal dysfunction. No variable reached statistical significance.

### 3.10. Factors Predicting of Necrosis

In the univariate analysis, age, male gender, past history of pancreatitis, cause of pancreatitis, and total Ranson did not reach statistical significance (*P*  value > 0.25). Only APACHE II score at admission significantly correlated with necrosis (*P*  value—0.04), wherein the chances of substantial necrosis rose by 20% with each unit increase of APACHE II score.

## 4. Discussion

Acute severe pancreatitis is a serious illness that has a high probability of complications and significant mortality [9]. Because of its prognostic association, estimating the severity is an important clinical task once the diagnosis is established. Although they are diagnostic standards, serum amylase and lipase do not determine severity. Clinical assessment alone can overlook severe disease in many patients.

Different approaches have been used estimate severity. Serum markers, such as tumor necrosis factor (TNF), C-reactive protein (CRP), polymorphonuclear elastase, methemalbumin, and pancreatic ribonucleases, have been evaluated as predictors, but none has gained widespread acceptance [10–12].

Multivariable scoring systems are another strategy. The first numeric system was proposed by Ranson et al. in 1974 for acute alcohol-induced pancreatitis and remains the most commonly used system [13]. It has 11 parameters—5 that are evaluated at admission and 6 after 48 hours. An increasing score corresponds to an increasing risk of mortality. The sensitivity and specificity of this scoring system range from 50% to 80%.

More recently, the Acute Physiological Assessment and Chronic Health Evaluation (APACHE II) assessment and monitoring system has become popular, because it is more reliable [14–16]. A shortcoming of biochemical markers and multivariable scoring systems, however, as noted earlier is their inability to assess the extent of injury to the pancreas and peripancreatic tissues.

In 1985, Balthazar and colleagues became the first researchers to grade severity of pancreatitis based on CT findings [4, 7], which subsequently have shown good correlation between pancreatic parenchymal necrosis, length of hospitalization, development of complications, and death [17]. In a cohort of patients with severe acute pancreatitis who were treated surgically, mortality was approximately 13 times more likely in persons in whom sterile necrosis was present (*P*  value 0.012 OR 13.704) [18].

 We have attempted to refine the prognostic predictive power of pancreatic necrosis by CT scan by dividing the patients into 2 arbitrary groups based on percentage of necrosis: greater or less than 50%.

A similar approach has been taken by Mortele and colleagues, where they scored the presence of pancreatic necrosis only as “no necrosis,” “minimal necrosis,” or “substantial necrosis,” thereby eliminating the arguably unnecessary separate categorization of patients who have 30% to 50% necrosis and patients who have more than 50% necrosis, because no significant difference exists in morbidity and mortality between these 2 groups [19, 20]. It also would help in simpler classification of these patients in practical clinical situations. As noted earlier, group B, with more than 50% necrosis on CT scan, had a significantly different clinical course and a much higher morbidity and mortality rate. Total hospital stay and ICU stay also were higher in this group.

## 5. Conclusions

- The rate of mortality (5% versus 40%) was significantly higher in patients with higher degree of necrosis.
- Only APACHE II significantly correlated with the degree of necrosis, wherein the chances of substantial necrosis rose by 20% with each unit increase of APACHE II score.
- The rate of organ dysfunction also rose in patients with more than 50% necrosis of the pancreas. Other morbidities and variables that were related to hospital stay also increased in individuals with higher necrosis.
- APACHE II score could be employed and studied further prospectively to help identify patients with pancreatic necrosis.

## Figures and Tables

**Table 1:** Rate of mortality and organ dysfunction depending on the extent of pancreatic necrosis.

Variable	<50%	>50%	Total	*P* value
Mortality				
Yes	1	6	7	^ ∗^ **0.027** Fisher's
%	5	40	20	
No	19	9	28	
%	95	60	80	
Organ dysfunction				
Cardiovascular				
Yes	2	8	10	^ ∗^ **0.008**
%	10	53.3	28.6	
No	18	7.0	25.0	
%	90	46.7	71.4	
Renal				
Yes	4	5	9	0.450
%	20	33.3	25.7	
No	16	10.0	26.0	
%	80	66.7	74.3	
Respiratory				
Yes	6	10	16	^ ∗^ **0.044**
%	30	66.7	45.7	
No	14	5.0	19.0	
%	70	33.3	54.3	

**Table 2:** Hospital stay and rate of other morbidities depending on the extent of pancreatic necrosis.

Variable	<50%	>50%	Total	*P* value
Requiring ICU stay				
Yes	2	8.0	10.0	^ ∗^ **0.008**
%	10	53.3	28.6	
No	18	7	25	
%	90	46.7	71.4	
Total hospital stay				
Mean	14.8	35.3	23.2	^ ∗^ **0.004**
s.d.	8.04	28.5	21.5	
Range	3–35	7–106	3–106	
Special care unit stay				
Mean	5.1	11.8	7.9	^ ∗^ **0.006**
s.d.	4.4	8.9	7.3	
Range	0–18	2–32	0–32	
NPO duration				
Mean	9.0	22.0	14.1	^ ∗^ **0.008**
s.d.	6.6	19.2	14.3	
Range	2–21	2–68	2–68	
Presence of infection				
Yes	4	2	6	0.667
%	20	13.3	17.1	
No	16	13	29	
%	80	86.7	82.8	
Surgery done				
Yes	2	3	5	0.627
%	10	20	16.7	
No	18	12	30	
%	90	80	83.3	
Pseudocyst^†^				
Yes	10	7	17	0.689
%	55.6	70.0	60.7	
No	8	3	11	
%	44.4	30.0	39.3	
Need for readmission^†^				
Yes	6	6	12	0.243
%	33.3	60.0	42.9	
No	12	4	16	
%	66.7	40.0	57.1	

^†^Patients who died or were lost to follow-up excluded from the analysis for pseudocysts and need for readmission.

**Table 3:** Ranson and APACHE scores of the patients depending on the degree of pancreatic necrosis.

Variable	<50%	>50%	Total	*P* value
Total Ranson's score				
Median	4.0	4.67	4.35	**0.445**
s.d.	1.51	1.94	1.73	
Range	2–6	2–7	2–7	
APACHE score				
Mean	6.4	10.4	8.1	^ ∗^ **0.018**
s.d.	5.29	3.52	5.0	
Range	1–23	4–18	1–23	

## Abbreviations

FNA: Fine needle aspiration
CT: Computed tomography
APACHE: Acute Physiological Assessment and Chronic Health Evaluation.
